# *In vivo* Dominant-Negative Effect of an *SCN5A* Brugada Syndrome Variant

**DOI:** 10.3389/fphys.2021.661413

**Published:** 2021-05-28

**Authors:** Nicolas Doisne, Marta Grauso, Nathalie Mougenot, Michel Clergue, Charlotte Souil, Alain Coulombe, Pascale Guicheney, Nathalie Neyroud

**Affiliations:** ^1^INSERM, UMR_S 1166 ICAN, Paris, France; ^2^UMR_S 1166, Faculté de Médecine Pitié-Salpêtrière, Sorbonne Université, Paris, France; ^3^UMS_28, Sorbonne Université, Paris, France

**Keywords:** Brugada syndrome, Na_v_1.5, *SCN5A*, animal model, electrophysiology, AAV

## Abstract

Loss-of-function mutations in the cardiac Na^+^ channel α-subunit Na_v_1.5, encoded by *SCN5A*, cause Brugada syndrome (BrS), a hereditary disease characterized by sudden cardiac death due to ventricular fibrillation. We previously evidenced *in vitro* the dominant-negative effect of the BrS Na_v_1.5-R104W variant, inducing retention of wild-type (WT) channels and leading to a drastic reduction of the resulting Na^+^ current (*I*_*Na*_). To explore this dominant-negative effect *in vivo*, we created a murine model using adeno-associated viruses (AAVs).

**Methods:**

Due to the large size of *SCN5A*, a dual AAV vector strategy was used combining viral DNA recombination and *trans*-splicing. Mice were injected with two AAV serotypes capsid 9: one packaging the cardiac specific troponin-T promoter, the 5′ half of *hSCN5A* cDNA, a splicing donor site and a recombinogenic sequence; and another packaging the complementary recombinogenic sequence, a splicing acceptor site, the 3′ half of *hSCN5A* cDNA fused to the *gfp* gene sequence, and the SV40 polyA signal. Eight weeks after AAV systemic injection in wild-type (WT) mice, echocardiography and ECG were recorded and mice were sacrificed. The full-length *hSCN5A-gfp* expression was assessed by western blot and immunohistochemistry in transduced heart tissues and the Na^+^ current was recorded by the patch-clamp technique in isolated adult GFP-expressing heart cells.

**Results:**

Almost 75% of the cardiomyocytes were transduced in hearts of mice injected with hNa_v_1.5 and ∼30% in hNa_v_1.5-R104W overexpressing tissues. In ventricular mice cardiomyocytes expressing R104W mutant channels, the endogenous *I*_*Na*_ was significantly decreased. Moreover, overexpression of R104W channels in normal hearts led to a decrease of total Na_v_1.5 expression. The R104W mutant also induced a slight dilatation of mice left ventricles and a prolongation of RR interval and P-wave duration in transduced mice. Altogether, our results demonstrated an *in vivo* dominant-negative effect of defective R104W channels on endogenous ones.

**Conclusion:**

Using a *trans*-splicing and viral DNA recombination strategy to overexpress the Na^+^ channel in mouse hearts allowed us to demonstrate *in vivo* the dominant-negative effect of a BrS variant identified in the N-terminus of Na_v_1.5.

## Introduction

Brugada syndrome (BrS) is an inherited autosomal-dominant cardiac channelopathy with incomplete penetrance, characterized by a typical electrocardiographic (ECG) pattern showing an ST-segment elevation in the right precordial leads (V1–V3) and an increased risk of sudden cardiac death due to ventricular fibrillation in structurally normal hearts (Brugada et al., 2018). Mutations in the *SCN5A* gene, encoding the cardiac voltage-gated sodium channel Na_v_1.5, have been identified in around 25% of affected individuals (Watanabe and Minamino, 2015) and commonly reveal loss-of-function properties reducing the sodium current *I*_*Na*_ either by gating abnormalities, trafficking defects, or premature stop codons leading to haploinsufficiency (Wilde and Brugada, 2011).

Na_v_1.5 constitutes the α-subunit of the cardiac Na^+^ channel complex, which includes other transmembrane subunits and intracellular partners that participate in its expression and function (Abriel et al., 2015). Unlike potassium channel genes, which encode monomers associating in tetramers to constitute the functional channel, Na_v_1.5 channels were thought to be structured as single entities. It was thus unexpected to report Na_v_1.5 mutants with a dominant-negative effect on wild-type (WT) channels, as we and others did a few years ago (Keller et al., 2005; Clatot et al., 2012; Mercier et al., 2012; Hoshi et al., 2014; Pambrun et al., 2014; Wang et al., 2020). In these studies, a decrease of *I*_*Na*_ exceeding the 50% of current density expected in case of haploinsufficiency was indeed observed when co-expressing some mutants with WT channels in a 1:1 ratio to mimic patient heterozygosity (Keller et al., 2005; Clatot et al., 2012; Hoshi et al., 2014). For example, we have reported that co-expression of the BrS R104W mutant and WT channels in HEK293 cells caused a loss of 80% of *I*_*Na*_ compared to WT channels expressed alone and demonstrated that this dominant-negative effect was due to an interaction between R104W α-subunits retained in the endoplasmic reticulum and WT channels (Clatot et al., 2012). It was then established that Na_v_1.5 α-subunits form dimers through an interaction site located in the domain I-II linker, and that Na_v_1.5 channels not only interact but also gate as dimers (Clatot et al., 2017).

Animal and cellular models have been created to simulate BrS, including transgenic mice, canine heart preparations, transgenic pork, expression of mutant *SCN5A* in different cellular models and, more recently, induced pluripotent stem cell-derived cardiomyocytes (iPS-CM) (Sendfeld et al., 2019). Knowledge gained from numerous studies achieved using experimental models has contributed to our current understanding of the pathophysiological mechanisms involved in BrS. Nevertheless, each of these models has revealed inherent limitations, e.g., the lack of cardiac background in heterologous expression systems, time and cost required to generate transgenic animal models and immaturity of iPS-CM.

In this study, we aimed to develop a versatile animal model of BrS using adeno-associated Viruses (AAVs) injection into mice. During the last two decades, AAVs turned out to be useful tools in gene therapy (Kaplitt et al., 1994; Guggino and Cebotaru, 2020) for the reason that they are small non-pathogenic and non-replicative DNA viruses with tissue-specific tropism extremely efficient for targeting *in vivo* transgene delivery (Prasad et al., 2011). One limitation of the use of AAVs as vectors for gene delivery is their intrinsic small packaging capacity of 5 kb (Dong et al., 1996). Nevertheless, the development of a dual-vector *trans*-splicing approach allowed to overcome this package-capacity limit (Duan et al., 2000; Sun et al., 2000; Ghosh et al., 2008, 2011). In this approach, the cDNA of a large gene can be split into two parts at the level of an intron and separately packaged into two individual AAVs, which will recombine in host cells and will be spliced in a mature full-length mRNA (Duan et al., 2000; Sun et al., 2000; Ghosh et al., 2008, 2011).

Taking advantage of the ability of AAVs to concatemerize, confirmed in other contexts like skeletal muscle (Sondergaard et al., 2015) and retina (Trapani et al., 2014), we used the dual-AAV *trans*-splicing strategy to overexpress in mice hearts and characterize *in vivo* the Na_v_1.5-R104W mutant previously reported to display a strong dominant-negative effect *in vitro* (Clatot et al., 2012). Our results showed for the first time that this dual-AAV *trans*-splicing approach allows overexpression of the full human *SCN5A* gene in up to 75% of injected-mice heart cells. Importantly, we recorded a significantly decreased endogenous *I*_*Na*_ in cardiomyocytes overexpressing R104W mutant channels and a reduction of the total Na_v_1.5 expression, demonstrating *in vivo* the dominant-negative effect of this BrS mutation in Na_v_1.5 on endogenous wild type (WT) channels. The R104W mutant overexpression also induced a slight dilatation of mice left ventricles, confirming that impairment of *I*_*Na*_ may be responsible for early stages of heart failure. Altogether our results demonstrated that the use of AAVs to overexpress *SCN5A* mutants *in vivo* is a relevant approach to create a versatile and valuable animal model of BrS.

## Materials and Methods

### Plasmids and Vectors

The AAV vectors pAcTnT-S and pAcTnT-eGFP were described previously (Prasad et al., 2011) and kindly provided by Dr. B. A. French (Virginia University, United States). The human Na_v_1.5 sequence hH1a (RefSeq accession number NM_000335.4) was subcloned from plasmid pcDNA3.1-hH1a, a gift of Dr. H. Abriel (University of Bern, Bern, Switzerland). The 75-bp donor and 58-bp acceptor consensus sequences were subcloned from the chimeric intron of the pCI mammalian expression vector (Promega, Madison, WI, United States). The 288-bp alkaline phosphatase (AP) sequence [pAG71 plasmid (Ghosh et al., 2011)] was kindly provided by Dr. D. Duan (University of Missouri, Columbia, MO, United States). The helper and packaging plasmids pXX6 and pAAV2-9 were a kind gift of Dr. S. Benkhelifa-Ziyyat (Institute of Myology, Paris, France). All plasmids were purified with the NucleoBond^®^ EF kit (Macherey Nagel, Düren, Germany) and sequenced for unwanted mutations (GATC, Konstanz, Germany).

Residue 104 of Na_v_1.5 is highly conserved between species and among sodium channels (Clatot et al., 2012) and, as a general concern, human and murine cardiac sodium channel sequences share a high homology of 95%. We thus decided to overexpress the human *SCN5A*-gene sequence carrying the R104W BrS variant into mice.

### Design of the hNa_v_1.5 *Trans*-Splicing Constructs

All elements for AAV recombination and splicing were inserted at the exon 17–18 junction of Na_v_1.5 cDNA (hH1a isoform; RefSeq NM_000335.4) using overlap extension PCR cloning to create 5′ and 3′ Na_v_1.5 halves for separate cloning in AAV vectors. The hNa_v_1.5 *trans*-splicing construct was generated by inserting a chimeric intron from the pCI vector in Na_v_1.5 cDNA at the junction between exons 17 and 18. The 133-bp chimeric intron was amplified on the pCI vector using primers hH1a.ex17-pCI.forward and hH1a.ex18-pCI.reverse (Table 1). The PCR was done with Phusion High-Fidelity DNA polymerase (Finnzymes, Waltham, MA, United States) at a hybridization temperature of 56°C and 35 cycles. After purification, the amplified pCI chimeric intron was inserted by overlap extension PCR (Bryksin and Matsumura, 2010) in the Na_v_1.5 cDNA. The overlap extension PCR was done at a hybridization temperature of 65°C and 25 cycles. Finally, the Na_v_1.5-pCI chimeric intron plasmid was obtained by transforming *Escherichia coli* cells after digestion of the overlap-extension PCR amplicons by *DpnI*.

**TABLE 1:** Primer sequences.

Name	5′-3′ primer sequence
hH1a.ex17-pCI forward	CTGGGCACGGAGGAGGAGTCCAGCAAGCAGgtaagtatcaaggttacaagacagg ^(1)^
hH1a.ex18-pCI.reverse	CTCTGGGCCACCGGACACAGGCTGGGATTCctgtggagagaaaggcaaagtg ^(1)^
hH1a-iDO-AP forward	gggcttgtcgagacagagaagactcttgCGCAGGGCAGCCTCTGTCATC^(2)^
hH1a-AP-iAC reverse	cagtaagaccaataggtgcctatcagaaacgTGGAGGCCGAAAGTACATGTTTCGC^(2)^
R104W forward	GCAAGACCATCTTCTGGTTCAGTGCCACC ^(3)^
R104W reverse	GTGGCACTGAACCAGAAGATGGTCTTGCC ^(3)^
5′hH1a-iDO-AP forward	CCACCATGGCAAACTTCCTATTACCTCGGGG ^(4)^
5′hH1a-AP-iAC reverse	CGACGTCGACTGGAGGCCGAAAGTACATGTTTCGC ^(5)^
AP-iAC-3′hH1a forward	TGCTCTAGACGCAGGGCAGCCTCTGTCATCTCC ^(6)^
3′hH1a-iAC-AP reverse	TCCCCGCGGCACGATGGACTCACGGTCCCTGTCCG ^(7)^

*^(1)^Lower cases indicate pCI chimeric intron and upper cases Na_v_1.5 exon 17–18 junction sequences. ^(2)^Lower cases indicate intron splicing consensus and upper cases alkaline phosphatase sequences. ^(3)^Mutated nucleotide is underlined. ^(4)^The kozak consensus sequence is underlined. ^(5)^The Sal I restriction site is underlined. ^(6)^The Xba I restriction site is underlined. ^(7)^The Sac II restriction site is underlined.*

In a second step, a successful clone was used to insert a reverse/complement-oriented recombinogenic AP sequence inside pCI chimeric intron sequence in a manner to obtain a 75 bp donor and a 58 bp acceptor consensus sequence for intron splicing. A 288 bp AP sequence was amplified on pAG71 plasmid using primers: hH1a-iDO-AP.forward and hH1a-AP-iAC.reverse (Table 1). PCR amplification protocol was obtained with a hybridization temperature of 65°C and 25 cycles. Purified AP amplification was inserted by overlap extension PCR in the previously obtained Na_v_1.5-pCI chimeric intron plasmid using an insert/plasmid ratio of 1:250, a hybridization temperature of 65°C and 25 cycles. The *DpnI* digested overlap extension PCR amplification was used to transform *E. coli* cells to obtain a Na_v_1.5-WT plasmid containing the whole recombinogenic/splicing cassette at the exon 17–18 junction.

R104W site-directed mutagenesis was then achieved on the Na_v_1.5-WT recombinogenic/splicing cassette-containing plasmid using the kit QuikChange II XL (Stratagene, Santa Clara, CA, United States) with the complementary primers R104W.forward and R104W.reverse (Table 1) following the manufacturer’s instructions.

### Cloning of 5′ and 3′ AAV-hNa_v_1.5 Expression Vectors

The 5′ hNa_v_1.5 recombinogenic/splicing half part was amplified by PCR on the Na_v_1.5 recombinogenic/splicing cassette-containing plasmid, WT or R104W mutated, using primers 5′hH1a-iDO-AP.forward and 5′hH1a-AP-iAC.reverse (Table 1). The purified amplification product was first cloned in pCRBlunt vector using TOP10 chemically competent *E. coli* (Invitrogen). The 3,653-bp HindIII-SalI fragment from a positive clone was then subcloned in the HindIII-SalI digested pAcTnT-S vector, using SURE2 competent cells (Stratagene), to obtain the pAcTnT.5′Na_v_1.5WT or pAcTnT.5′Na_v_1.5R104W viral plasmids.

The 3′hNa_v_1.5 recombinogenic/splicing half part was amplified by PCR on the Na_v_1.5 recombinogenic/splicing cassette-containing plasmid using primers AP-iAC-3′hH1a.forward and 3′hH1a-iAC-AP.reverse (Table 1). The purified amplification product was first cloned in pCRBlunt vector using TOP10 chemically competent *E. coli*. The 3172-bp XbaI-SacII digested fragment from a positive clone was then subcloned in the XbaI-SacII digested pAcTnT-eGFP vector, using SURE2 competent cells, to obtain the pA.3′Na_v_1.5-eGFP viral plasmid. Absence of insert recombination during bacterial amplification was verified by digestion pattern of restriction sites SmaI and MscI in AAV inverted terminal repeat (ITR) sequence.

### Production and Titration of AAV Particles

Adeno-associated viruses were either in-house prepared or produced by the Viral Vector Core of Nantes University (France). We used the triple transfection of HEK293T cells with pXX6 and pAAV2-9 as helper plasmids respectively to obtain AAVs of serotype-9 capsids. Viral:helper plasmid molar ratio for HEK293T transfection was 1:1 and a total of 145 μg of DNA was used to transfect 500 square centimeter of sub-confluent HEK293T cells with polyethylenimine (PEI) as transfecting agent in 2%-fetal calf serum DMEM media. Cells were collected 3 days after transfection to recover AAV particles. AAVs were purified after ammonium-sulfate precipitation (∼50% saturation) and Benzonase (Sigma-Aldrich, United States) digestion of free DNA by ultracentrifugation on iodixanol gradient. Viral particles were finally concentrated using Vivaspin 100 kD columns (Sartorius, Göttingen, Germany).

To assess viral genome titer, we performed the Universal Real-Time PCR with AAV2 ITR specific primers and probe as described previously (Aurnhammer et al., 2012) but using the LightCycler 480 probes master kit (Roche, Basel, Switzerland) in a final reaction volume of 20 μl. PCR mix contained a final concentration of 0.5 μM of each primer and 0.1 μM of probe and 2 μl of template. PCR protocol consisted in one denaturation of 15 min at 95°C, 45 amplification cycles of 1 min at 95°C and 1 min at 60°C and a final cooling cycle of 10 s at 40°C. Viral template was added in four 1:10 serial dilutions from 10^–2^ to 10^–5^ of purified stock. For standard curve, we used six serial dilutions of viral vector pAcTnT-eGFP, *NdeI* linearized, containing from 5.8 10^7^ to 5.8 10^2^ copy number per μl. The viral vector pAcTnT-eGFP stock concentration was determined with Quant-iT^TM^ PicoGreen^®^ dsDNA reagent and kit (Molecular Probes, United States).

### Animals

Three to five-days old C57Bl6/J mice were injected through one jugular vein with a maximum of 100 μl of either AAV-cTnT-eGFP or a mix of AAV-cTnT-5′hNa_v_1.5 (WT or R104W) and AAV-3′hNa_v_1.5-eGFP. Depending on the titer of AAV preparations, we injected between 2.8 10^11^ and 1.45 10^12^ viral particles for AAV-cTnT-eGFP and between 6.84 10^11^ and 2.6 10^12^ total viral particles for the mix of AAV-cTnT-5′hNa_v_1.5 and AAV-3′hNa_v_1.5-eGFP in a 1:1 molar ratio. Seven weeks after injection, ECG and echocardiography were performed. Mice were then sacrificed and the heart was excised for *ex vivo* and *in vitro* experiments.

### Echocardiography and Surface ECG Measurements

Echocardiography was performed on lightly anesthetized 8-week-old mice under isoflurane. Non-invasive measurements of left ventricular dimensions were evaluated using echocardiography-Doppler (Vivid 7 Dimension/Vivid7 PRO; GE Medical System Co., Vélizy, France) with a probe emitting ultrasounds from 9- to 14-MHz frequency. The two-dimensionally guided Time Motion mode recording (parasternal long-axis view) of the left ventricle (LV) provided the following measurements: diastolic and systolic septal (IVSd and IVSs) and posterior wall thicknesses (LVPWd and LVPWs), internal end-diastolic diameter (LVEDD) and end-systolic diameter (LVESD), and heart rate. Each set of measurements was obtained from the same cardiac cycle. At least three sets of measurements were obtained from three different cardiac cycles. Fractional shortening was calculated by the following formula: [(LVEDD–LVESD)/LVEDD] × 100. Cardiac output was measured by Pulse Wave Doppler using the following formula: [(πd^2^/4) × VTI × HR], where d is the diameter of aorta, VTI, the subaortic velocity time integral and HR the heart rate.

Surface ECG measurements were also performed under isoflurane anesthesia on 8-week-old mice. Two-lead ECGs were recorded with 29-gauge subcutaneous electrodes on a computer using an analog-digital converter (iox 2.4.2.6; emka Technologies, Paris, France) for monitoring and analyzed with ecgAUTO software (emka Technologies, Paris, France). Recordings were filtered at 50 Hz, and a stable signal was reliably obtained before proceeding. ECG traces were signal averaged and analyzed for heart rate (RR interval), P wave, PR and QRS interval duration.

### Total Protein Extraction and Western-Blot

Total proteins were extracted from frozen pieces of hearts from mice injected with either AAV-GFP or the mix of Na_v_1.5 AAVs, in lysis buffer (50 mM Tris pH 7.5, 150 mM NaCl, 2 mM EDTA, 1% Triton and complete protease inhibitor cocktail from Roche) for 1.5 h at 4°C on a wheel. The soluble fractions from 30-min centrifugation at 13,000 × *g* (4°C) were then used for western blot experiments.

Total extracted proteins were separated on a 3–8% acrylamide SDS-PAGE gel and transferred to a nitrocellulose membrane. After the membrane was cut horizontally between 250 and 130 kD, and vertically on the molecular weight, it was incubated with primary antibodies followed by infrared IR-Dye secondary antibodies (LI-COR Biosciences, United States). Primary antibodies used were as follows: rabbit anti-GFP (1:2000, Torrey Pines Biolabs, United States), rabbit anti-Na_v_1.5 (1:200, Alomone Labs, Israel) and mouse anti-α-tubulin (1:1000, Sigma-Aldrich, United States). Proteins were detected using the Odyssey Infrared Imaging System (LI-COR Biosciences, United States). Signals were quantified using ImageJ software. Total protein signals were normalized to α-tubulin levels in hearts expressing GFP as a control of mice injection.

### Immunohistochemistry and Imaging

Indirect immunofluorescence was performed on 10 μm control (GFP) Na_v_1.5-WT or R104W-injected mouse ventricle cryosections fixed with paraformaldehyde for 15 min. Sections were washed twice for 5 min with phosphate buffer saline (PBS), blocked in PBS-5% bovine serum albumin (BSA) for 30 min at room temperature. Sections were then incubated overnight with primary antibodies at 4°C: the rabbit anti-GFP (1:2000, Torrey Pines Biolabs, United States) to detect exogenous hNa_v_1.5-GFP, and mouse anti-α-actinin 2 (1:500, Sigma-Aldrich, United States). Heart sections were then washed twice with PBS and incubated 1 h with secondary antibodies: goat anti-rabbit Alexa Fluor 488 and goat anti-mouse Alexa Fluor 594 (1:1000, Molecular Probes, Thermo Fisher Scientific, United States), and the nuclear dye DAPI (1:2000, Merck, Germany) diluted in the blocking buffer. Control experiments were performed by omitting primary antibodies.

Labeled ventricle sections were observed with a DeltaVision epifluorescent microscope (20× or 60×). Images were analyzed with DeltaVision imaging system (GE Healthcare, Seattle, WA, United States) equipped with 3D-deconvolution. For each sample, series of consecutive plans were acquired (sectioning step: 0.2 μm).

### Cardiomyocyte Isolation

Eight- to ten-week-old mice were anesthetized and heart was quickly excised. After cannulation of the aorta, hearts were mounted to a constant pressure Langendorff system. First, hearts were rinsed for around 4 min by perfusion of a free-Ca^2+^ Tyrode solution (see composition below in Solutions) containing 10 mM BDM (ButaneDione Monoxime) and 20 mM Taurine and subsequently perfused with enzymatic solution containing 0.09 mM Ca^2+^ Tyrode with 3 mg/ml collagenase type 2 (Worthington Biochemical Corporation, Lakewood, NJ, United States) for 6–10 min. Hearts were then immersed in a 0.18 mM Ca^2+^ Tyrode containing 5 mg/ml BSA and ventricles were cut into small pieces and further dissociated into single cells by gentle shaking. The Tyrode Ca^2+^ concentration was then two times doubled every 5 min to reach 0.72 mM. Cells were kept in this 0.72 mM Ca^2+^ Tyrode solution and used within 5 h after isolation.

### Electrophysiology

Patch-clamp recordings were carried out at room temperature (22 ± 1°C). Ionic currents were recorded by the whole-cell patch-clamp technique with the amplifier VE-2 (Alembic, Canada). Patch pipettes (Corning Kovar Sealing code 7052, WPI) had resistances of 1–1.5 MΩ. Currents were filtered at 5 kHz (23 dB, 8-pole low-pass Bessel filter) and digitized at 30 kHz (NI PCI-6251, National Instruments, Austin, TX, United States). Data were acquired and analyzed with ELPHY software (G. Sadoc, CNRS, Gif/Yvette, France). To measure peak *I*_*Na*_ amplitude and determine current–voltage (*I/V* curves) and activation-*Vm* relationships, currents were elicited by test potentials of 0.2 Hz frequency to −100 to +60 mV for 50 msec by increments of 5 or 10 mV from a holding potential of −120 mV. The steady-state inactivation-*Vm* protocol was established from a holding potential of −120 mV and a 2 s conditioning pre-pulse was applied in 5 or 10 mV increments between −140 and +20 mV, followed by a 50 msec test pulse to −20 mV at 0.2 Hz frequency. Data for the activation-*Vm* and steady-state availability-*Vm* relationships of *I*_*Na*_ were fitted to the Boltzmann equation: *Y* = 1/{1 + exp[-(*Vm*-*V*_1/2_)/*k*]}, where *Vm* is the membrane potential, *V*_1/2_ is the half-activation or half-availability potential, *k* is the slope factor and *Y* represents the relative conductance.

### Solutions

Composition of free-Ca^2+^ Tyrode solution was (in mM): 135 NaCl, 4 KCl, 2.5 MgCl_2_, 10 HEPES, 10 glucose, pH 7.4 (NaOH). Cells were bathed in an extracellular Tyrode solution containing (in mM): 135 NaCl, 4 KCl, 2.5 MgCl_2_, 2 CaCl_2_, 10 glucose, 10 HEPES, pH 7.4 (NaOH). Patch pipette medium was (in mM): 135 CsCl, 1 CaCl_2_, 2 MgCl_2_, 4 Mg-ATP, 15 EGTA, 10 HEPES, pH 7.2 (CsOH). During current recording, cells were perfused with an external solution with reduced Na^+^ concentration containing (in mM): 10 NaCl, 123.5 CsCl, 2 CaCl_2_, 2.5 MgCl_2_, 10 HEPES, 10 glucose, 20 Tetra Ethyl Ammonium, 3 4-AP, 3 CoCl_2_, pH 7.4 (CsOH).

### Statistical Analysis

Data are represented as mean ± SEM. Statistical significance was estimated with GraphPad Prism^®^ software (San Diego, CA, United States) by Student *t*-test after population normality, checked by Shapiro–Wilk test, was assessed in each group. *P* < 0.05 was considered significant.

## Results

### Cloning Strategy to Produce Hybrid Dual Vectors Allowing for Overexpression of the Full-Length *hSCN5A* Gene in Mouse Heart Tissue

One limitation of the use of AAVs as vectors for transgene delivery is their intrinsic small packaging capacity of 5 kb (Dong et al., 1996), which is less than the full-length *SCN5A* cDNA size (6048 bp). To overcome this restriction, we developed in this study a dual-vector *trans*-splicing approach permitting to double AAVs’ package-capacity limit, as previously published (Duan et al., 2000; Sun et al., 2000; Ghosh et al., 2008, 2011). In this approach, after host infection by both AAV populations and viral genome unpackaging, the transgene cDNA is reconstituted thanks to a short highly recombinogenic sequence and its excision from the pre-mRNA through intron splicing.

**FIGURE 1:**
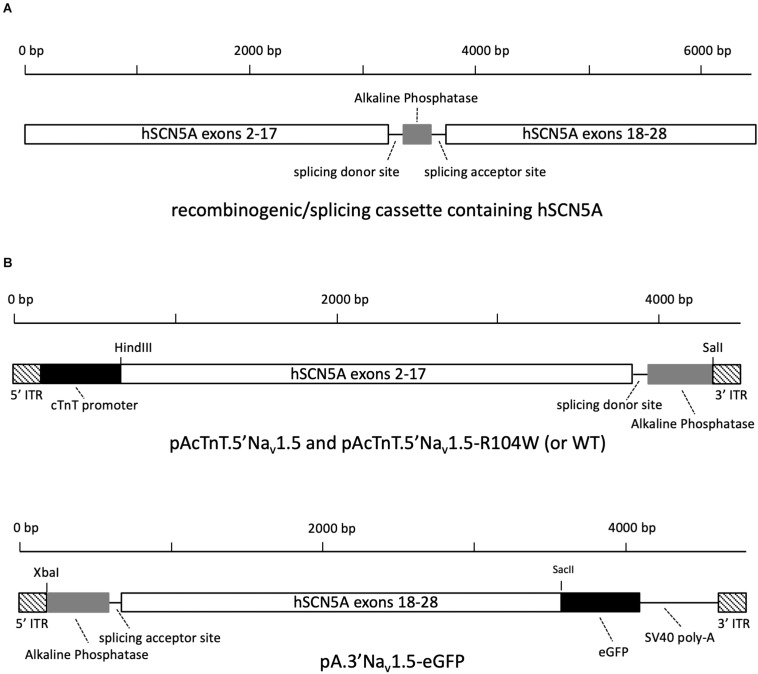
Design of 5′ and 3′ AAV-hNa_v_1.5 expression vectors. **(A)** The recombinogenic/splicing cassette inserted at the junction between exons 17 and 18 of *SCN5A* cDNA contains splicing donor and acceptor sequences from the chimeric intron of pCI vector and a reverse-complementary 288 bp sequence from human placental alkaline phosphatase. **(B)** Schematic representation of plasmids pAcTnT.5′Nav1.5-R104W and pA.3′Nav1.5-eGFP cloned to produce two distinct *trans*-splicing AAV populations.

To this end, we first chose a suitable region in *SCN5A* to insert a DNA cassette composed of the first third of the human AP sequence (Ghosh et al., 2011) with splicing donor and acceptor consensus sites from the chimeric intron of pCI vector located at its 5′ and 3′ ends (Figure 1A). Using the Human Splicing Finder tool^1^, we obtained the best score for the junction between exons 17 and 18 of *SCN5A*, which also permitted to cut the full cDNA into two portions of equivalent size and to obtain dual hybrid AAV genomes of 4.4 and 4.7 kb, respectively. To avoid appearance of cloning restriction sites, we inserted the recombinogenic/splicing cassette by overlap extension PCR cloning, a technique allowing for insertion of large sequences (Bryksin and Matsumura, 2010).

With the aim to restrain *SCN5A* overexpression to heart tissue, we combined the use of AAV serotype 9 for its tropism for heart tissue and the chicken cardiac troponin T (cTnT) promoter for its cardiac specificity (Prasad et al., 2011), to design viral genome plasmids. Also, in order to quantify heart-tissue viral transduction and *trans*-splicing efficiency and to visualize cardiomyocytes overexpressing human Na_v_1.5 channels during patch-clamp recordings, we fused the sequence of eGFP to the 3′-end of *SCN5A*.

As cloning final results, we obtained the AAV plasmids named pAcTnT.5′hH1a and pAcTnT.5′hH1a-R104W containing the hNa_v_1.5 or hNa_v_1.5-R104W cDNA from ATG to nucleotide 3228 (exon 2–17), splicing donor site and AP sequences, and the AAV plasmid named pA.3′hH1a-eGFP carrying the AP, splicing acceptor site and hNa_v_1.5 cDNA from nucleotide 3229–6045 (exon 18 to 28 with deletion of the stop codon) sequences as shown on Figure 1B. We also designed pAcTnT-eGFP for control experiments.

### Overexpression of Human Nav1.5-R104W Channels in Mouse Heart Tissue Using Dual *Trans*-Splicing AAVs

**FIGURE 2:**
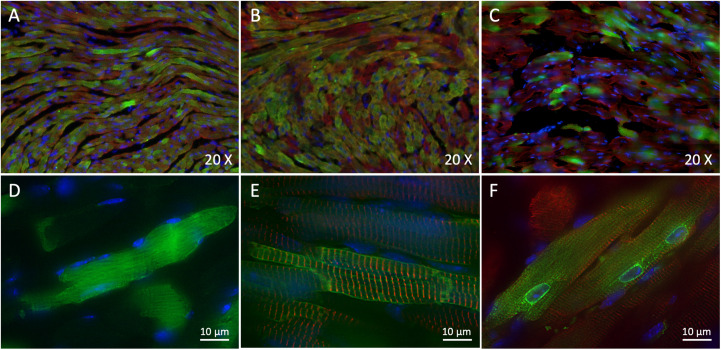
*Trans*-splicing dual AAVs efficiently transduce heart tissue. **(A,D)** Representative 3-dimensional deconvolution images of GFP (green) and α-actinin 2 (red) immunostaining of 10 μm GFP-injected mouse heart cryosections. **(A)**: 20×; **(D)**: 60×; scale bar: 10 μm. **(B,E)** Representative 3-dimensional deconvolution images of hNa_v_1.5 (green) and α-actinin 2 (red) immunostaining of 10 μm hNa_v_1.5-injected mouse heart cryosections. **(B)**: 20×; **(E)**: 60×; scale bar: 10 μm. **(C,F)** Representative 3-dimensional deconvolution images of hNav1.5-R104W (green) and α-actinin 2 (red) immunostaining of 10 μm R104W-injected mouse heart cryosections. **(C)**: 20×; **(F)**: 60×; scale bar: 10 μm. Exogenous hNa_v_1.5-GFP channels were stained in green by the anti-GFP antibody, and nuclei in blue using DAPI. Note that AAV-GFP transduced approximately 75% of injected mice cardiomyocytes, as did the *trans*-splicing AAVs expressing hNa_v_1.5, while the hNav1.5-R104W mutant channels were observable in around 1/3 of heart cells. The GFP protein was expressed in the whole cytoplasm of transduced cells, whereas the hNa_v_1.5 channels were expressed at the cell surface and hNa_v_1.5 channels carrying the R104W variant were mostly retained in the perinuclear area of cardiomyocytes.

To assess whether our strategy of dual vectors was efficient to express the full-length human Na_v_1.5-GFP channel in mouse cardiac tissue and to compare its efficiency to single-AAV transduction, we first made 10 μm cryosections of injected-mouse ventricles to study expression and localization of hNa_v_1.5-GFP or GFP alone by immunohistochemistry. As shown on Figure 2, we observed a strong expression of GFP in ∼75% of heart cells in AAV-GFP injected mouse cardiac ventricles (Figures 2A,D) and an equally important expression (∼75%) of hNa_v_1.5-WT in ventricular cardiomyocytes of mice injected with dual *trans*-splicing vectors AAV-cTnT-5′Na_v_1.5-WT and AAV-3′hNa_v_1.5-eGFP (Figures 2B,E). A robust but lower expression of hNa_v_1.5 channels (∼30%) was observable in mice injected with hNa_v_1.5-R104W dual AAVs (Figures 2C,F).

It is noteworthy that GFP overexpressed alone was localized in the whole cytoplasm (Figure 2D), while hNa_v_1.5-WT channels were mainly expressed at the cell surface (Figure 2E). This was not the case for hNa_v_1.5-R104W channels retained in the perinuclear area of heart cells, suggesting a retention of the mutant sodium channels in endoplasmic reticulum (Figure 2F), as we already observed in HEK293 cells (Clatot et al., 2012).

### Cardiac Functional Effects of Overexpressing the Dominant-Negative Variant hNa_v_1.5-R104W in Mice

**FIGURE 3:**
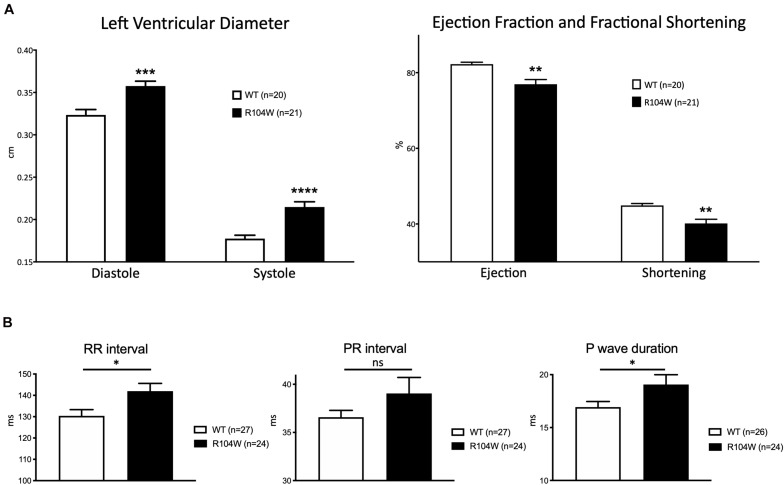
Cardiac functional effects of the dominant-negative mutant R104W in Na_v_1.5. **(A)** Left ventricular diameter (in cm), left ventricular ejection fraction and fractional shortening (in %) measured by echocardiography in GFP-injected (*n* = 20) and Na_v_1.5-R104W-injected mice (*n* = 21). Overexpression of the hNa_v_1.5-R104W channel induced a significant left ventricular dilation combined to a significant reduction of left ventricular ejection fraction and fractional shortening. **(B)** RR interval, PR interval, and P wave duration (in ms) measured on ECG recordings in control (*n* = 27) and hNa_v_1.5-R104W-injected mice (*n* = 24). Overexpression of the hNa_v_1.5-R104W mutant channel led to a slight but significant prolongation of RR interval and P wave duration. PR interval was also slightly increased in R104W overexpressing mice when compared to controls, but not significantly. *: *P* < 0.05; **: *P* < 0.005; ***: *P* < 0.001; ****: *P* < 0.0001.

Our first concern was to verify that AAV systemic injection in newborn mice had no consequences on heart function assessed by cardiac echocardiography. We compared echocardiography parameters in non-injected and AAV-GFP injected mice and observed no significant differences (Supplementary Table 1). We thus considered AAV-GFP-injected mice as the control group in our study. On the other hand, overexpression of the R104W mutated sodium channel significantly increased left ventricular diameter (0.32 ± 0.006 cm in WT, *n* = 20 vs. 0.36 ± 0.006 cm in R104W, *n* = 21 for diastolic diameter, *P* < 0.001 and 0.18 ± 0.004 cm in WT, *n* = 20 vs. 0.21 ± 0.006 cm in R104W, *n* = 21 for systolic diameter, *P* < 0.0001) and decreased left ventricular ejection fraction (82 ± 0.5 % in WT, *n* = 20 vs. 77 ± 1.2 % in R104W, *n* = 21, *P* < 0.005) and fractional shortening (45 ± 0.5 % in WT, *n* = 20 vs. 40 ± 1.1 %, *n* = 21 in R104W, *P* < 0.005) when compared to control mice, as shown in Figure 3A. Moreover, overexpression of hNa_v_1.5-R104W channels significantly increased end-diastolic (EDV) and stroke volumes (SV) compared to WT (Table 2). Altogether these data suggest that overexpression of the R104W mutant sodium channel in mouse heart leads to early stages of dilated cardiomyopathy.

**TABLE 2:** Echocardiographic parameters.

	HR (bpm)	dIVS (cm)	sIVS (cm)	dPWT-LV (cm)	sPWT-LV (cm)	EDV (ml)	ESV (ml)	SV (ml)	CO (l/min)
**WT (*n* = 20)**	572 ± 6	0.056 ± 0.001	0.091 ± 0.003	0.06 ± 0.002	0.1 ± 0.003	0.089 ± 0.005	0.016 ± 0.001	0.073 ± 0.004	0.042 ± 0.002
**R104W (*n* = 21)**	556 ± 10	0.059 ± 0.002	0.091 ± 0.003	0.061 ± 0.002	0.1 ± 0.003	0.12 ± 0.005***	0.026 ± 0.002***	0.091 ± 0.004**	0.05 ± 0.003*

Control GFP-injected (WT) and hNa_v_1.5-R104W-injected mice (R104W). HR: Heart Rhythm; dIVS: diastolic Intra-Ventricular Septum; sIVS: systolic Intra-Ventricular Septum; dPWT-LV: diastolic Posterior Wall Thickness-Left Ventricle; sPWT-LV: systolic Posterior Wall Thickness-Left Ventricle; EDV: End-Diastolic Volume; ESV: End-Systolic Volume; SV: Stroke Volume; CO: Cardiac Output. *: *P* < 0.05; **: *P* < 0.005; ***: *P* < 0.001.

To evaluate the effects of the dominant-negative BrS variant R104W overexpression on mice ECG parameters, we recorded surface ECG in injected mice (Figure 3B and Table 3) and observed a small but significant reduction of heart rate (RR interval: 130 ± 2.9 ms in WT, *n* = 27 vs. 142 ± 3.6 ms in R104W, *n* = 24, *P* < 0.05) and a significant prolongation of the P wave duration (17 ± 0.5 ms in WT, *n* = 26 vs. 19 ± 0.9 ms in R104W, *n* = 24, *P* < 0.05). We observed no other significant differences in ECG parameters between both groups, while PR interval slightly prolonged in R104W overexpressing mice (37 ± 0.7 ms in WT, *n* = 27 vs. 39 ± 1.7 ms in R104W, *n* = 24) (Table 3).

**TABLE 3:** Electrocardiographic parameters.

	RR interval (ms)	P wave duration (ms)	PR interval (ms)	QRS interval (ms)	QT interval (ms)
WT	130 ± 3 (*n* = 27)	17 ± 0.5 (*n* = 26)	37 ± 0.7 (*n* = 27)	13 ± 0.2 (*n* = 27)	54 ± 1.6 (*n* = 12)
R104W	142 ± 4 (*n* = 24)*	19 ± 0.9 (*n* = 24)*	39 ± 1.7 (*n* = 24)	13 ± 0.3 (*n* = 16)	53 ± 1.1 (*n* = 5)

Control GFP-injected (WT) and hNa_v_1.5-R104W-injected mice. *: *P* < 0.05.

### *Ex vivo* Consequences of hNa_v_1.5 Overexpression in Transduced Mouse Cardiomyocytes

**FIGURE 4:**
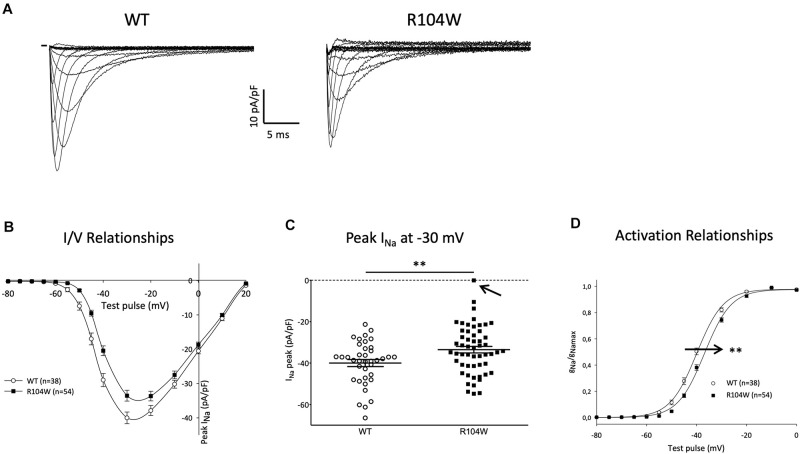
Overexpression of the hNa_v_1.5-R104W mutant decreased endogenous *I*_*Na*_. **(A)** Typical Na^+^ currents recorded in control (WT, 10 mM Na^+^ outside) and hNa_v_1.5-R104W-injected mouse cardiomyocytes (R104W, 10 mM Na^+^ outside). **(B)** I/V relationships of peak Na^+^ current recorded in control (WT, *n* = 38) and hNa_v_1.5-R104W-injected mouse cardiomyocytes (R104W, *n* = 54). **(C)** Distribution of peak Na^+^ current recorded at –30 mV in control (WT, *n* = 38) and hNa_v_1.5-R104W-injected mouse cardiomyocytes (R104W, *n* = 54). Note that, in one cell indicated by an arrow, *I*_*Na*_ was null. **(D)** Activation/voltage relationships of peak Na^+^ current in control (WT, *n* = 38) and hNa_v_1.5-R104W-injected mouse cardiomyocytes (R104W, *n* = 54). **: *P* < 0.005. Overexpression of the hNa_v_1.5-R104W mutant significantly decreased *I*_*Na*_ recorded in injected mouse and shifted activation relationship to more positive potentials.

Since hNav1.5-R104W overexpression in mice hearts using AAV dual vectors seemed to cause a cardiac dysfunction, we then sought to record the sodium current in cardiomyocytes isolated from injected mice hearts. We recorded the global *I*_*Na*_ resulting from co-expression of endogenous WT murine channels and hNav1.5-R104W channels by the patch-clamp technique in whole cell configuration (Figure 4). In hNa_v_1.5-R104W-expressing cardiomyocytes, the *I*_*Na*_ peak amplitude at −30 mV was significantly decreased by 15 % when compared to control cardiomyocytes (−40 ± 1.7 pA/pF in WT, *n* = 38 vs. −34 ± 1.6 pA/pF in R104W, *n* = 54, *P* < 0.05) as shown in Figures 4A,B, suggesting an *in vivo* dominant-negative effect of this BrS variant. In Figure 4C, we represented all peak *I*_*Na*_ amplitude recorded in each cell to highlight cellular variation of *I*_*Na*_ in the R104W group: from 0 (indicated by an arrow) to −55 pA/pF.

As shown on Figure 4D, activation curve of R104W overexpressing cells was significantly shifted to more positive potentials by 3 mV, compared to controls (V_1/2_ = −39.9 ± 0.7 mV in WT, *n* = 38 vs. −36.9 ± 0.6 mV in R104W, *n* = 54, *P* < 0.005), suggesting a loss-of-function of mutant channels, as it was already shown in transfected HEK293 cells (Clatot et al., 2012). No significant difference was observed in inactivation curves (V_1/2_ = −77 ± 1.1 mV in WT, *n* = 33 vs. −76.3 ± 0.7 mV in R104W, *n* = 42).

In order to demonstrate that our strategy of dual AAVs was efficient to modulate the endogenous sodium current, we also recorded *I*_*Na*_ in cardiomyocytes isolated from mice overexpressing hNa_v_1.5-WT. We observed a significant and important increase (65 %) of total *I*_*Na*_ in AAV-hNav1.5-WT injected hearts (Supplementary Figure 1). Altogether our electrophysiological results suggest that the use of the Troponin T cardiac-specific promoter to drive Na_v_1.5 overexpression together with a dual AAV vector approach was powerful to modulate the murine *I*_*Na*_.

### Human Na_v_1.5-R104W Overexpression Reduced Total Na_v_1.5-Protein Expression

**FIGURE 5:**
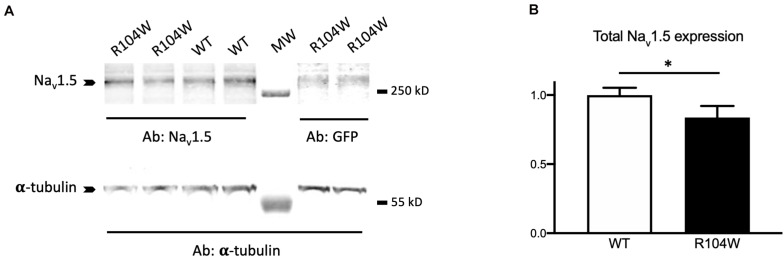
hNa_v_1.5-R104W overexpression decreased the Na_v_1.5-protein total quantity. **(A)** Representative western blot of total cardiac proteins extracted from GFP (WT) or R104W injected mice. Total Na_v_1.5 proteins were revealed using the anti-Na_v_1.5 antibody and normalized to α-tubulin levels in hearts expressing GFP as a control of AAV injection and expression. **(B)** Total Na_v_1.5 protein expression was significantly decreased in R104W injected mice (*n* = 12), when compared to control ones (*n* = 10). *: *P* < 0.05.

To confirm the *in vivo* dominant-negative effect of Na_v_1.5-R104W BrS variant, we explored the possible explanation of this effect at the cellular level by quantifying the expression of the total Na_v_1.5 protein in injected-mouse hearts on western-blots (Figure 5 and Supplementary Figure 2 for raw data). We observed that the quantity of total Na_v_1.5 protein, revealed by a specific anti-Na_v_1.5 antibody, was decreased in hNa_v_1.5-R104W-injected mouse hearts compared to controls (*P* < 0.05; Figure 5B), suggesting a degradation of endogenous murine channels.

## Discussion

Although several BrS-causing *SCN5A* mutations have been characterized using cellular and animal models, integrated understanding of the mechanisms linking sodium channel dysfunction to cardiac pathophysiology is still lacking. With the aim to develop a versatile and ready-for-use BrS animal model of *SCN5A* variant characterization, we used AAVs to generate a powerful system of overexpression of a large gene targeted to mice heart tissue. We demonstrated that *in vivo* expression of a human BrS *SCN5A* variant was responsible for a dominant-negative effect, which confirmed what was previously observed *in vitro*. Indeed, hNa_v_1.5-R104W cardiac overexpression in mice decreased global *I*_*Na*_ and total Na_v_1.5-protein expression, prolonged PR interval and P-wave duration, and led to early stages of dilated cardiomyopathy.

### Development of Dual Hybrid AAV Vectors for Cardiac-Targeted Expression of Na_v_1.5

Taking advantage of a technique published in 2000 (Duan et al., 2000; Sun et al., 2000), we developed dual hybrid AAV vectors carrying the Troponin T cardiac-specific promoter and the full-length *SCN5A*-gene sequence fused to the eGFP reporter gene, to overexpress Na_v_1.5 variants in their physiological cardiac background. The challenge was to overcome limited cargo capacity of AAVs and to design vectors capable of recombination and *trans*-splicing to reconstitute the full-length *SCN5A*-eGFP sequence, once in mice cardiac cells.

This strategy proof-of-principle was established by Dr. Duan’s group in 2007 in a study designed to assess mice whole-body transduction using *trans*-splicing AAVs (Ghosh et al., 2007, 2008). Then, dual AAV hybrid vectors have been exploited in human gene therapy in the past years, demonstrating the interest of this technique for *in vivo* efficient gene transfer (Koo et al., 2014; Trapani et al., 2014; Trapani, 2018; Barbon et al., 2021). To the best of our knowledge, we present here, for the first time, overexpression of a cardiac large gene in ∼75% of mice cardiomyocytes. Most importantly, our results demonstrated that dual AAVs could be used to create animal models mimicking human diseases. If the use of the cTnT promoter confirmed a robust cardiomyocyte-specific expression of the transgene (Prasad et al., 2011), we also believe that systemic injection of AAVs in the early stages of life (3–5 days after birth) helped to reduce mice immune response and to favor cell transduction efficiency (Ghosh et al., 2007; Hu et al., 2010).

A limitation of our strategy was the choice to design a protein fusion of Na_v_1.5 and eGFP. Indeed, the dominant-negative effect of the R104W variant implied retention and degradation of Na_v_1.5 channels, leading to degradation of the GFP and loss of its reporter gene function. If in GFP and hNa_v_1.5-injected mouse cardiomyocytes, green positive cells were easy to visualize for patch-clamp recordings, native fluorescence was almost not visible in hNa_v_1.5-R104W overexpressing cardiomyocytes, and we could not select the transduced cells to record. This was most probably responsible for the large variation of currents represented in Figure 4C, and likely for an underestimation of the *in vivo* dominant-negative effect of R104W on endogenous WT channels. Nevertheless, it is worth to note that one *I*_*Na*_ recorded in a hNa_v_1.5-R104W-injected mouse cardiomyocyte (indicated by an arrow in Figure 4C) was null, even if recorded with 135 mM Na^+^ in the outer solution. We suspect the degradation of mutated channels to lead also to the apparent lower transduction rate observed for hNa_v_1.5-R104W AAVs compared to hNa_v_1.5-WT ones (Figures 2B,C). Nevertheless, we believe that the hNa_v_1.5-R104W-transduction rate was underestimated as a result of GFP degradation, since the transduction rate of hNa_v_1.5-WT reached 75%, with the exact same dual AAV genome, except for the R104W missense mutation. On another hand, immunostaining of the GFP fused to the channel allowed to localize mutant channels and to confirm that hNa_v_1.5-R104W was overexpressed in injected-mice cardiomyocytes (Figures 2C,F) and in injected-mice cardiac tissues analyzed in western-blots (Figure 5A), an observation that would have not been possible using a reporter gene not fused to the channel.

### Comparison With Other Animal Models of Brugada Syndrome

A mouse model with targeted disruption of *Scn5a* has been established in 2002 (Papadatos et al., 2002). If homozygous knock out (KO) mouse embryos die during mid-gestation due to structural abnormalities of the heart, heterozygous mice show normal survival and several cardiac electrical defects such as decreased atrial, atrioventricular, and ventricular conduction and increased susceptibility to pacing-induced ventricular arrhythmias (Papadatos et al., 2002; van Veen et al., 2005). Despite being detectable in less than a half of mice cardiomyocytes, the R104W dominant-negative variant induced a prolongation of RR interval, P wave duration and PR interval as a consequence of the significant decrease of *I*_*Na*_, like observed in Scn5a^+/–^ mice (Leoni et al., 2010). However, we did not observe any prolongation of QRS intervals, compared to transgenic deficient mice. It is worth to note that we recorded a small but significant rightward shift of R104W activation curve in transduced mice cardiomyocytes, as in HEK293 cells (Clatot et al., 2012), accounting for the loss-of-function characteristics of this variant.

Two knock in (KI) models of *SCN5A* mutants have been developed (Sendfeld et al., 2019): one in mice, exhibiting an overlap syndrome whose conduction-defect severity was strain-dependent (Remme et al., 2006), and one in pig, showing prolonged P and QRS wave duration and prolonged PR intervals, consistent with slowed cardiac conduction (Park et al., 2015). Both KI transgenic models, like our model of overexpression using viral vectors, suggest that expressing a specific mutation led to a particular phenotype, recapitulating the complexity of BrS.

Overexpression of *SCN5A* in transgenic mice has been shown to shorten P wave duration and PR interval, while QRS and QT intervals remained unchanged (Zhang et al., 2007). This was consistent with the observation that mice overexpressing *SCN5A* exhibit accelerated atrioventricular, atrial, and ventricular conduction (Liu et al., 2015). It was therefore not surprising to record an increase of the P wave duration and the PR interval, even if not significant, in our model of overexpression of a dominant-negative Na_v_1.5 mutation. At this stage, we can hypothesize that action potential upstroke velocity (dV/dt) is impaired in R104W-overexpressing mice since their cardiac *I*_*Na*_ is significantly decreased, but further experiments of action potential recordings should be realized to confirm this hypothesis.

We chose to develop our dual AAVs strategy in the mouse since several other genetically modified mouse models were available to compare our results with (Derangeon et al., 2012; Sendfeld et al., 2019) and because mouse is the most utilized mammal in scientific research for its size and similarities with human. Nevertheless, further studies should be conducted to adapt this approach to bigger animals with features closer to human cardiac physiology. Moreover, our model, as others, has inherent limitations, as it is a model of overexpression using a promoter chosen to drive a robust cardiac specific expression, but which did not allow to control the transgene expression level.

### BrS Variants in *SCN5A* and Dilated Cardiomyopathy

It has long been assumed that cardiac structural abnormalities are undetectable in patients with loss of-function *SCN5A* channelopathies, in coherence with the conventional concept that Na_v_1.5 is only involved in maintaining cardiac electrical integrity. However, this paradigm has been challenged in the last years as loss-of-function *SCN5A* mutations are found in a growing number of patients with dilated cardiomyopathy (Zaklyazminskaya and Dzemeshkevich, 2016; Asatryan, 2019), by the demonstration that Nav1.5 is part of a macromolecular complex which contains cytoskeleton proteins (Rook et al., 2012) and by the observation of dilatation and impairment in ventricular contractile function in patients carrying loss-of-function BrS *SCN5A* variants (van Hoorn et al., 2012). As recently reviewed by Rivaud et al. (2020), an alternative concept is emerging in which Na_v_1.5 may also be involved in maintaining cardiac structural integrity by non-ionic mechanisms. In the light of this analysis, we can hypothesize that impairment of *I*_*Na*_ may be responsible for cardiac dilatation and early stages of heart failure.

### Possible Mechanisms of the *in vivo* Dominant-Negative Effect of hNav1.5-R104W

In the strict sense of the term, a dominant-negative effect is observed when a decrease of *I*_*Na*_ exceeding the 50% of current density expected in case of haploinsufficiency is recorded while co-expressing mutants with WT channels in a 1:1 ratio to mimic patient heterozygosity. As discussed above, degradation of the reporter protein fused to the mutated channel very likely underestimated the mutant functional effects on *I*_*Na*_. Considering this limitation, we understand the significantly reduced *I*_*Na*_ and the decrease of total Na_v_1.5 protein expression in R104W-injected mice as an evidence of the *in vivo* dominant-negative effect of R104W. At this stage, we can only speculate that WT endogenous Na_v_1.5 channels degradation occurred through their interaction with R104W α-subunits, as shown previously *in vitro* (Clatot et al., 2012). This hypothesis was further supported by the abnormal perinuclear localization of mutant channels, compared to WT-overexpressed ones (Figures 2E,F). Nevertheless, further studies should be conducted to demonstrate the interaction between hNa_v_1.5-R104W and mNa_v_1.5 endogenous channels as the mechanism of the *in vivo* dominant-negative effect of R104W mutant channels.

## Conclusion

To summarize, our results showed for the first time that a dual-AAV *trans*-splicing approach allows overexpression of a large gene encoding an ion channel in up to 75% of injected-mice cardiomyocytes. Applied to overexpression of a BrS variant in mouse heart, this strategy enabled us to confirm *in vivo* the R104W variant dominant-negative effect previously observed *in vitro*. Altogether our results demonstrated that the use of AAVs to overexpress *SCN5A* mutants *in vivo* is a relevant approach to create a versatile and valuable animal model of BrS. Furthermore, the success of our approach of dual *trans*-splicing AAVs to overexpress *SCN5A* in the heart constitutes the proof-of-concept of future work aimed at developing novel treatment for malignant arrhythmias observed in *SCN5A* loss-of-function-related channelopathies.

## Data Availability Statement

The raw data supporting the conclusions of this article will be made available by the authors, without undue reservation.

## Ethics Statement

The animal study was reviewed and approved by Comité d’éthique en expérimentation animale Charles Darwin N°5 INSERM & Sorbonne Université.

## Author Contributions

ND and MG realized all the experiments of molecular biology and electrophysiology. ND, MG, and NN analyzed the data. NM recorded echocardiographies and ECGs. MC produced some AAV preparations. CS performed western blots. ND, MG, AC, PG, and NN contributed to manuscript writing. PG and NN funded the project. All authors contributed to the article and approved the submitted version.

## Conflict of Interest

The authors declare that the research was conducted in the absence of any commercial or financial relationships that could be construed as a potential conflict of interest.

## Footnotes

1 http://www.umd.be/HSF/
