# Supplementary material for: In vivo Dominant-Negative Effect of an SCN5A Brugada Syndrome Variant
Source: Front Physiol. 2021 May 28;12:661413. doi: 10.3389/fphys.2021.661413 (PMC8195286; doi:10.3389/fphys.2021.661413)
Supplement: Supplementary file 1 [file Presentation_1.PPTX]

## Slide 1
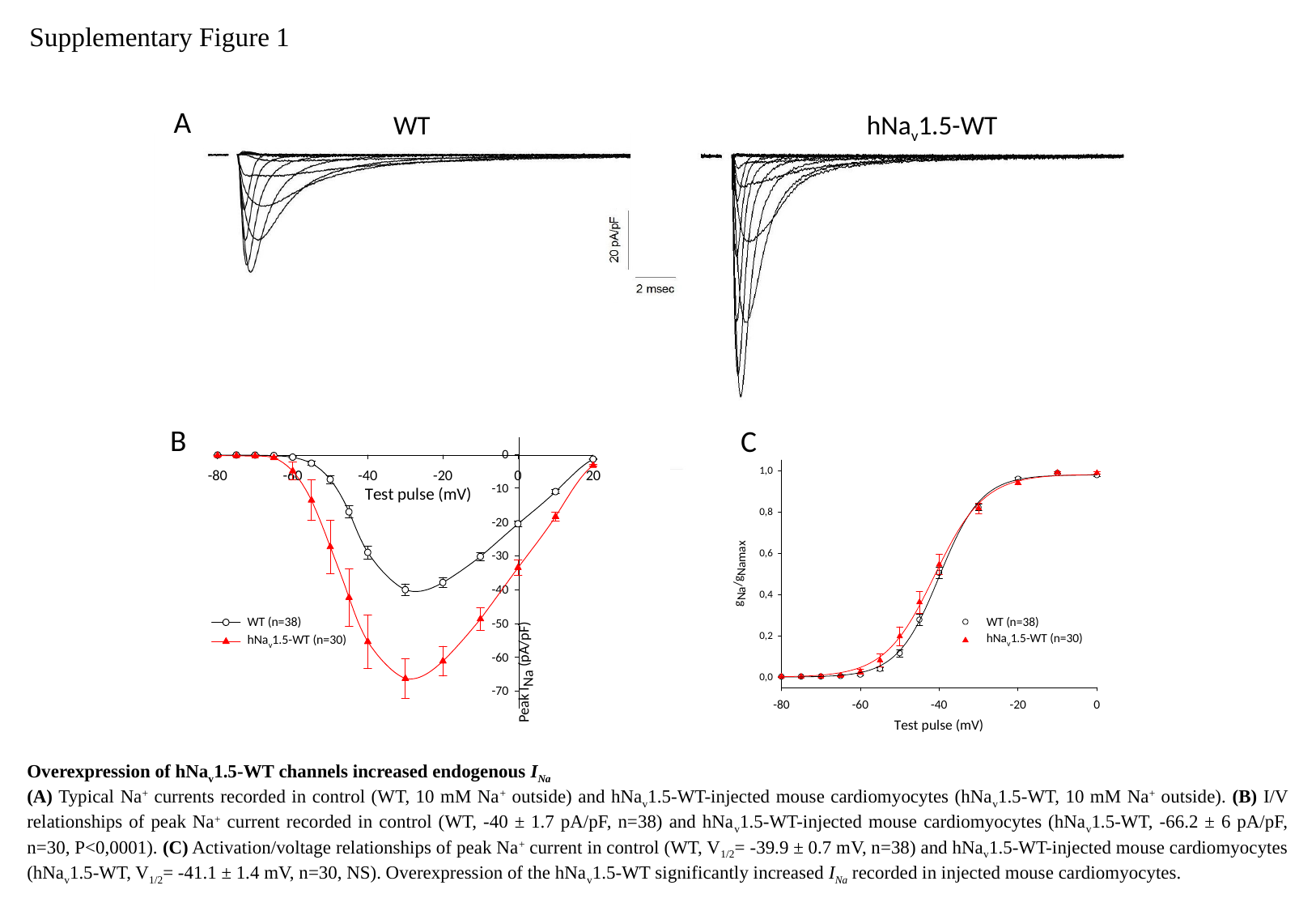

Supplementary Figure 1
A
WT
hNav1.5-WT
B
C
Overexpression of hNav1.5-WT channels increased endogenous INa
(A) Typical Na+ currents recorded in control (WT, 10 mM Na+ outside) and hNav1.5-WT-injected mouse cardiomyocytes (hNav1.5-WT, 10 mM Na+ outside). (B) I/V relationships of peak Na+ current recorded in control (WT, -40 ± 1.7 pA/pF, n=38) and hNav1.5-WT-injected mouse cardiomyocytes (hNav1.5-WT, -66.2 ± 6 pA/pF, n=30, P<0,0001). (C) Activation/voltage relationships of peak Na+ current in control (WT, V1/2= -39.9 ± 0.7 mV, n=38) and hNav1.5-WT-injected mouse cardiomyocytes (hNav1.5-WT, V1/2= -41.1 ± 1.4 mV, n=30, NS). Overexpression of the hNav1.5-WT significantly increased INa recorded in injected mouse cardiomyocytes.

## Slide 2
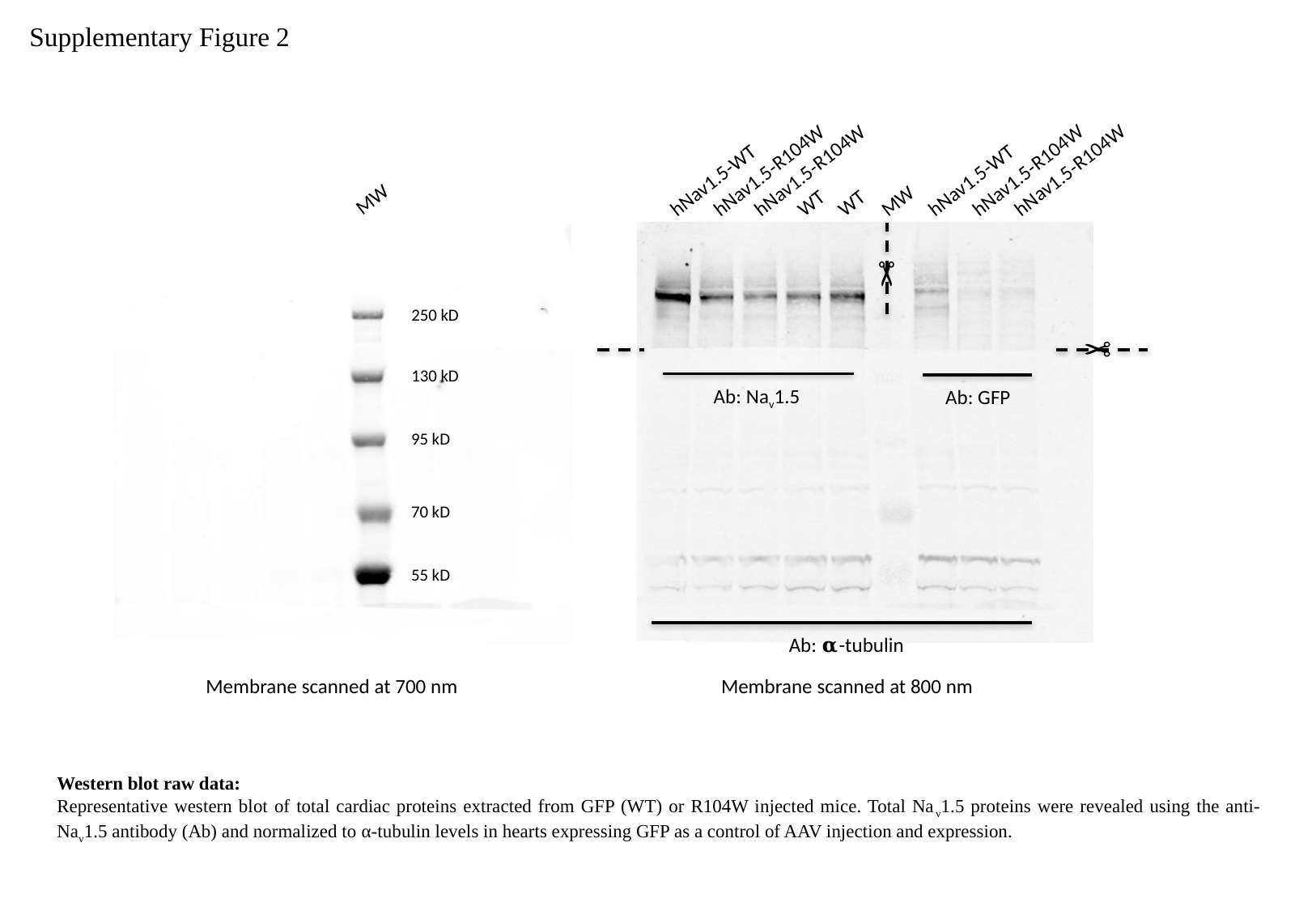

Supplementary Figure 2
hNav1.5-R104W
hNav1.5-R104W
hNav1.5-R104W
hNav1.5-R104W
hNav1.5-WT
hNav1.5-WT
MW
MW
WT
WT
✂︎
250 kD
✂︎
130 kD
Ab: Nav1.5
Ab: GFP
95 kD
70 kD
55 kD
Ab: 𝛂-tubulin
Membrane scanned at 700 nm
Membrane scanned at 800 nm
Western blot raw data:
Representative western blot of total cardiac proteins extracted from GFP (WT) or R104W injected mice. Total Nav1.5 proteins were revealed using the anti-Nav1.5 antibody (Ab) and normalized to α-tubulin levels in hearts expressing GFP as a control of AAV injection and expression.

## Slide 3
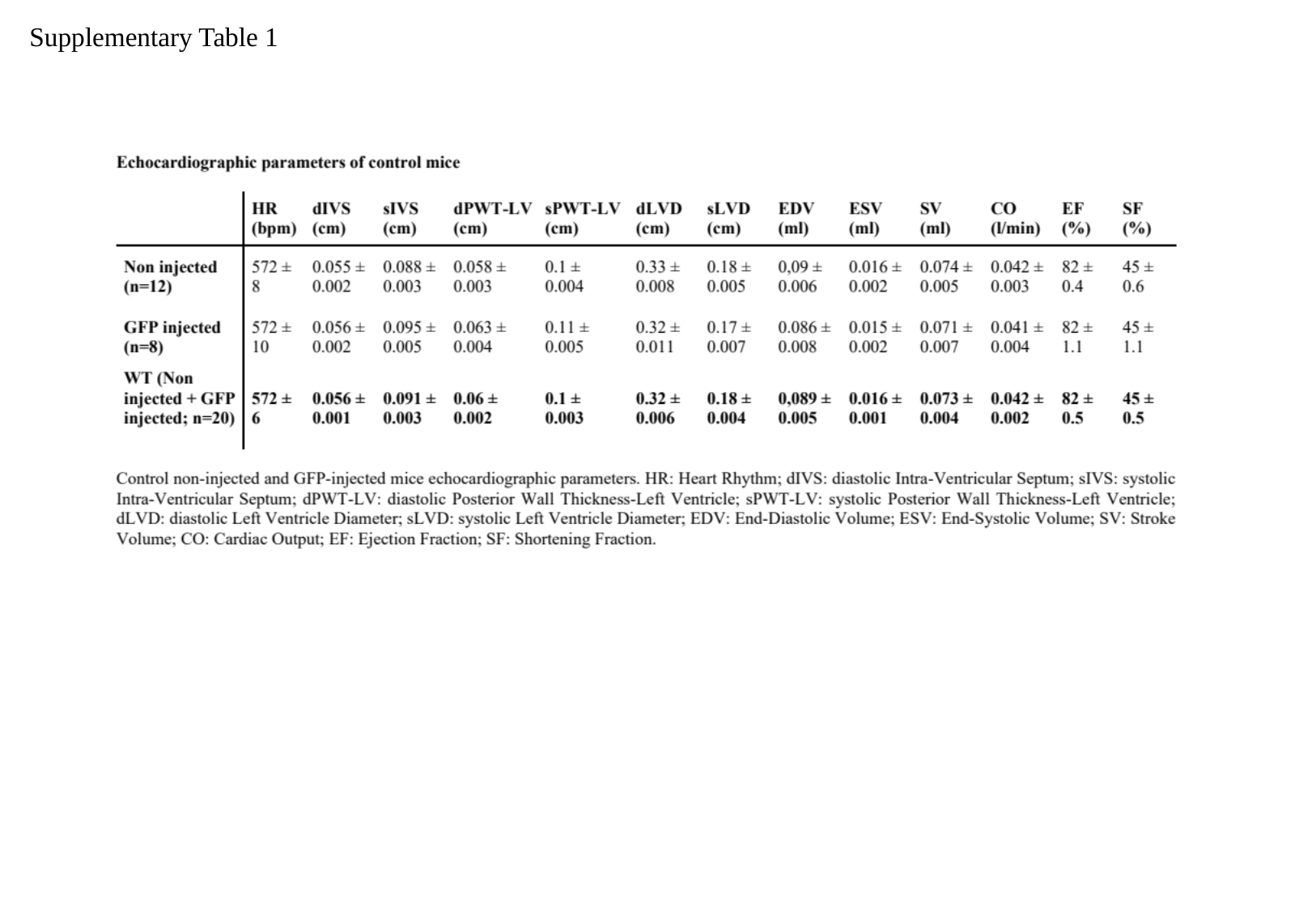

Supplementary Table 1
